# A bibliometric analysis of research on pediatric preoperative anxiety (2007–2022)

**DOI:** 10.3389/fped.2024.1327118

**Published:** 2024-03-25

**Authors:** Yue Zhong, Huishu Gong, Feiyu Long, Xingchen Zhou, Jun Zhou, Maohua Wang, Tao Peng

**Affiliations:** ^1^ Department of Anesthesiology, The Affiliated Hospital, Southwest Medical University, Luzhou, Sichuan, China; ^2^ Anesthesiology and Critical Care Medicine Key Laboratory of Luzhou, The Affiliated Hospital, Southwest Medical University, Luzhou, Sichuan, China

**Keywords:** children, preoperative anxiety, bibliometric analysis, hot topics, research frontiers

## Abstract

**Objective:**

This study aimed to analyze the current state of research on preoperative anxiety in children through CiteSpace, VOSviewer, and the identification of hot spots and frontiers.

**Method:**

Relevant data were retrieved from the Web of Science Core Collection using the search terms children and preoperative anxiety. Data were analyzed using VOSviewer (version 1.6.18), CiteSpace (5.7. R5) software, and Scimago Graphica.

**Results:**

A total of 622 articles were published between 2007 and 2022, with an increasing trend over time. Kain, Zeev N. (13; 2.09%) and Dalhousie University (15; 2.41%) were the most influential authors and most prolific institutions, respectively. The United States (121; 19.45%) was the country with the most publications. Pediatric anesthesia (55; 8.84%) had the most publications. High-frequency keywords were categorized into three themes, including nonpharmacologic interventions for preoperative anxiety in children, preoperative medications, and risk factors for anxiety; of these, “predictor” (38; 2016) and “sedative premedication” (20; 2016) were the most studied keywords over the past 6 years. “Distraction” (67; 2019) and “dexmedetomidine” (65; 2019) have been the main areas of interest in recent years.

**Conclusion:**

Research on preoperative anxiety in children has been the focus of increasing attention over the past fifteen years, with the majority of publications from high-income countries. This review provides a useful perspective for understanding research trends, hot topics, and research gaps in this expanding field.

## Introduction

Surgery is undoubtedly an extremely unpleasant mental experience for children undergoing elective surgery, especially during the induction of anesthesia. It is estimated that 50% of children suffer from anxiety before surgery (1), and children with higher levels of preoperative anxiety tend to require deeper intraoperative anesthesia as well as more pain medication compared to others. In addition, children are more likely to suffer from separation anxiety and maladaptive behaviors during the recovery phase of the postoperative period (2) and are thought to be associated with a higher incidence of delirium during the emergence phase (3). To mitigate a number of adverse effects of anxiety, previous researchers and clinical practitioners have explored a variety of methods to reduce preoperative anxiety, and there are two main categories of interventions to reduce preoperative anxiety: sedative medications and nonpharmacological interventions (4) (also known as psychobehavioral interventions). Among them, the use of preoperative sedative medication is considered a reliable strategy to reduce preoperative anxiety but has limitations in its application; first, many children experience adverse side effects such as nausea and vomiting (5). Second, the use of anxiolytic medications may be limited by a long onset and duration of action, and it is often difficult to determine the optimal time to administer these medications in a busy surgical setting. Third, there is an increased risk of adverse postoperative outcomes associated with the use of preoperative sedative medications. These include delirium, agitation, and pain; delayed awakening from anesthesia in patients undergoing ultrashort procedures; and delayed discharge from the hospital (3, 6, 7). Given the adverse effects of pharmacologic treatments, nonpharmacologic interventions are becoming more popular, and nonpharmacologic interventions include clown doctors, virtual reality, cognitive behavioral therapy, music therapy, hypnosis, guided imagery relaxation therapy, massage, games (8–13).Although there have been several published studies on preoperative anxiety, confirming that a variety of interventions have a positive impact on reducing preoperative anxiety, the number of studies is small, and the heterogeneity of sample sizes, research methodologies and study designs means that further research is needed, while there is still a lack of quantitative bibliometric analysis in the field of preoperative anxiety. Therefore, this paper uses bibliometric research methods to organize and summarize the research in this field over the past fifteen years to quantitatively show the development path, research hotspots, and research trends in this research field and to provide a more comprehensive compendium and analysis of the development of this field through knowledge mapping.

Bibliometrics is a method of quantitative analysis of publications using mathematical and statistical methods (14). Bibliometrics takes literature as the object of study and studies the distribution, flow, evaluation and evolution of literature. It quantifies and analyzes literature by mathematical and statistical methods, combining the theories and methods of computer science, sociology, intelligence and other disciplines. The main objects of its measurement are: volume of literature, issuing authors/institutions/regions/countries, citations, co-citations, keywords and so on. The most essential feature of bibliometrics is that its output must be “quantity”. Literature quantity is the basic concept in bibliometrics, which is used to describe the quantity of literature in a specific field or topic, and through the statistical analysis of the literature quantity, we can understand the active degree of research and development trend in this field. By analyzing the issuing authors, institutions, regions and countries, we can understand the research cooperation and the distribution of research power in this field. Citation analysis refers to the method of analyzing the citation relationship between academic literature. Through citation analysis, we can understand the influence of a particular literature, the academic exchange in a research field and the mutual influence between disciplines. Co-citation analysis refers to the method of analyzing the number of times two or more pieces of academic literature are jointly cited by other literature. Through co-citation analysis, we can understand the degree of correlation between these pieces of literature, and then reveal the intrinsic connection between disciplines. Keyword analysis refers to the method of classifying and identifying the topics of academic literature. Through the analysis of keywords, we can understand the research hotspots and cutting-edge issues in a particular field, as well as the intersections and development trends between disciplines. Bibliometrics is chosen because it reflects the quantity, quality and influence of literature in a more systematic way. This method helps to analyze the development trend of literature, disciplinary frontiers, hot content, author cooperation and influencing factors, etc. It can be used in academic research, disciplinary construction, scientific and technological assessment and information services, etc. Secondly, it can help experts and knowledge managers in the institution to find literature and information with higher relevance with the help of citation and keyword information of literature in the bibliometric method to improve the efficiency of knowledge acquisition. Some studies have shown that medicine in general is at the forefront of knowledge development in bibliometrics (15), in short, the trend of literature production in bibliometrics in medical applications is positive. Various forms of visualization software have been created, such as CiteSpace, VOSviewer, and HistCite, to help scholars construct knowledge graphs, evaluate the latest cutting-edge research advances, and visualize trends in scientific publications (16). In this study, two bibliometric analysis software programs, CiteSpace and VOSviewer, were used to construct visual maps to visualize valuable information hidden under indexed details such as authors, institutions, countries, journals, keywords, or any other data.

## Methods

### Data source and search strategy

Relevant publications for the period 2007–2022 were collected from the Science Citation Index Expanded (SCI-EXPANDED) and the Social Science Citation Index (SSCI) in the Web of Science Core Collection (WoSCC) (17), which provided high-quality publications on a variety of scientific disciplines from around the world (18).Web of Science (Core Collection) was chosen as the data source for this study because Web of science, as a high-quality digital literature resource database, has been accepted by many researchers as the most comprehensive and authoritative database suitable for bibliometric analysis (19). The retrieval sentence was determined as follows: TS = (Preoperative Period OR Period, Preoperative) AND TS = (Child OR Children) AND TS = (Anxiety OR Angst OR Social Anxiety OR Anxieties, Social OR Anxiety, Social OR Social Anxieties OR Hypervigilance OR Nervousness OR Anxiousness). The study restriction type was “article or review”, and the initial subject search yielded 131 records. We expanded the data set by citation indexing and found 1,204 citations to original research articles and English-language review articles between 2007 and 2022. After CiteSpace duplicate removal and manual screening, 622 records were identified and used in subsequent analyses. Data were extracted from the included publications, exported in the “Plain text file” format, and then recorded as “Full record and cited references”. The language of the documents was limited to English. The search strategy is shown in Supplementary Figure S1.

## Data analysis

For bibliometric analysis in this study, two visualization and analysis software programs were used, CiteSpace and VOSviewer. CiteSpace, developed by Chaomei Chen, is a Java-based visualization software for bibliometric analysis that is completely free (16, 20). In this study, CiteSpace software version 5.7.R5 was used. It was used to analyze the collaborative network of countries, authors, institutions, keywords, and cocited references to reveal the structure of one or more domains, and the resulting visual network graph consisted of many nodes and lines. Nodes represent different elements, such as country/region, institution, author, and cocited reference. The size of an individual node reflects the number of items observed. The thickness of each circle indicates the number of citations relative to a “time zone” or time frame, and the lines between nodes indicate collaboration/co-occurrence/cocitation of articles.

VOSviewer can be used to create bibliometric visualization charts, which can be used to build author or journal maps based on paper data or keyword maps based on co-occurrence data. Unlike commonly used bibliometric software, VOSviewer focuses on the graphical representation of bibliometric content, which is certainly an easy way for researchers to understand, and its benefits are especially evident in large-scale research efforts (21). The advantages of VOSviewer are particularly evident in the measurement of large studies. The main objective of using VOSviewer is to analyze the bibliometric network and construct visual graphs, ultimately gaining insight into research hotspots and trends in the field of study. We downloaded the records retrieved by WoSCC, converted these data into plain text format for export, including full records and citations, and finally imported them into VOSviewer and CiteSpace for bibliometric and visualization analysis. In this study, VOSviewer was first used to chart the annual number of publications in childhood preoperative anxiety. Next, we used VOSviewer to visualize the distribution of research on pediatric preoperative anxiety over time in each country, as well as authors’ co-citation relationships and interdisciplinary collaborations in the field, while VOSviewer was used to statistically derive the distribution of research in the field across major institutions and journals. Finally, keywords related to pediatric preoperative anxiety were clustered according to their citation frequency. Based on the clustering results, the hotspots in the field were obtained, and the nodes were also colored according to the year of publication of the keywords so that the changes of the hotspots over time could be visualized. Then we used CiteSpace to create a global visual map of the growth of the literature on a subject basis. And then the software was used to visualize the co-cited reference network and the cluster analysis network, with purple circles for literature with centrality >0.1, and the top five articles with high centrality were counted and analyzed. Finally, the keywords and cited documents in the co-citation network were detected by CiteSpace, and the strength of their citation emergence was ranked, and the keywords and documents with the strongest citation emergence imply that the relevant research has recently received a high degree of attention. It is beneficial to understand the most active research topics and research frontiers, and at the same time, these influential articles may become the basis for future research.

Scimago Graphica was used to visualize the collaborative network between countries/regions is more intuitive than the collaborative network of countries/regions generated by VOSviewer, which shows the strength of the linkage between countries’ postings, while the latter visualizes the publication of studies on pediatric preoperative anxiety in each country.

## Results

### Bibliometric analysis of publication years

**Figure 1:**
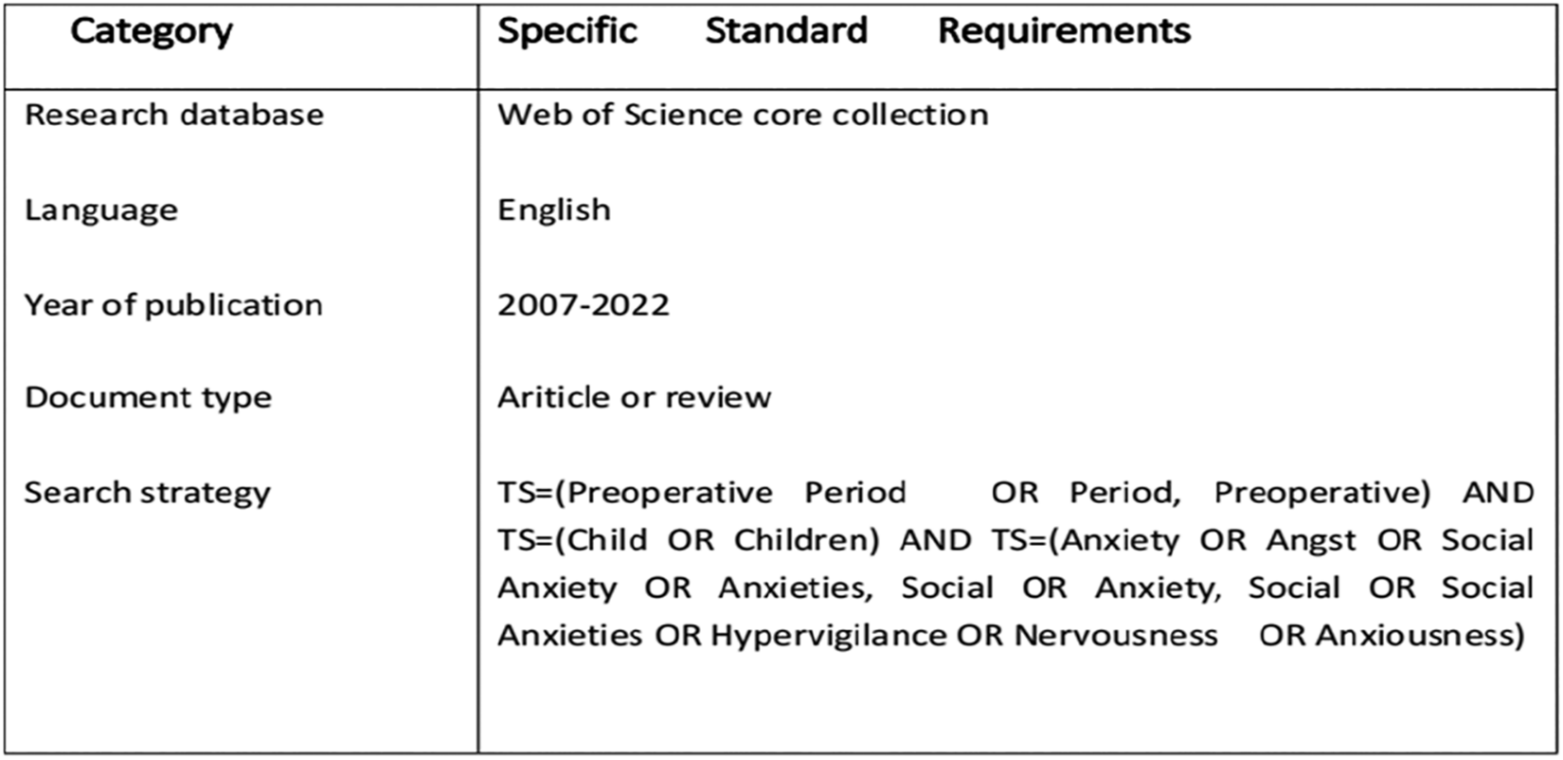
Data sources.

The 622 papers used in this study come from 54 countries, 986 institutions, and 2,817 authors, published in 253 journals and citing 14,200 citations from 4,378 journals. Figure 1 shows the temporal distribution of publications in this area of research, which shows overall growth. Although the annual number of publications in 2019 is slightly lower than that in the previous two years, the number of publications in 2022 is by far the most productive year in the last 15 years, with 103 publications, and all articles have stabilized at 40 or more since 2017, suggesting that this area of research is attracting the attention of an increasing number of scholars. In addition, a growth trend model coefficient of determination (*R*^2^) = 0.9654 was conducted, which showed a significant correlation between the year of publication and the number of publications.

### Bibliometric analysis of countries and institutions

To understand which countries have the most prominent contribution to the field of preoperative anxiety in children, this study analyzed the number of articles published by 54 countries. First, countries with a number of articles greater than or equal to 3 were visualized by Vosviewer, and the results are shown in Figure 3A, where the larger the round nodes are, the more articles are published. The connecting lines of the nodes indicate the strength of the association, and the coarser the connecting lines indicate that more two countries cooperate to publish articles. The node color represents the time of sending. Figure 3A and Table 1 show that the distribution of countries in this area is very uneven. The United States conducted the largest number of studies on preoperative anxiety (121; 19.45%), accounting for both the first studies and the largest proportion of studies. China followed (112; 18.01%), accounting for the largest proportion of late studies; developed countries such as Canada, Germany, Australia, and France began studying preoperative anxiety before developing countries such as China and India. Figure 3B shows the collaborative networks between countries/regions and shows that the five countries with the highest centrality were Australia (0.77), the United Kingdom (0.34), Germany (0.20), China (0.19), and Canada (0.11).

**Figure 2:**
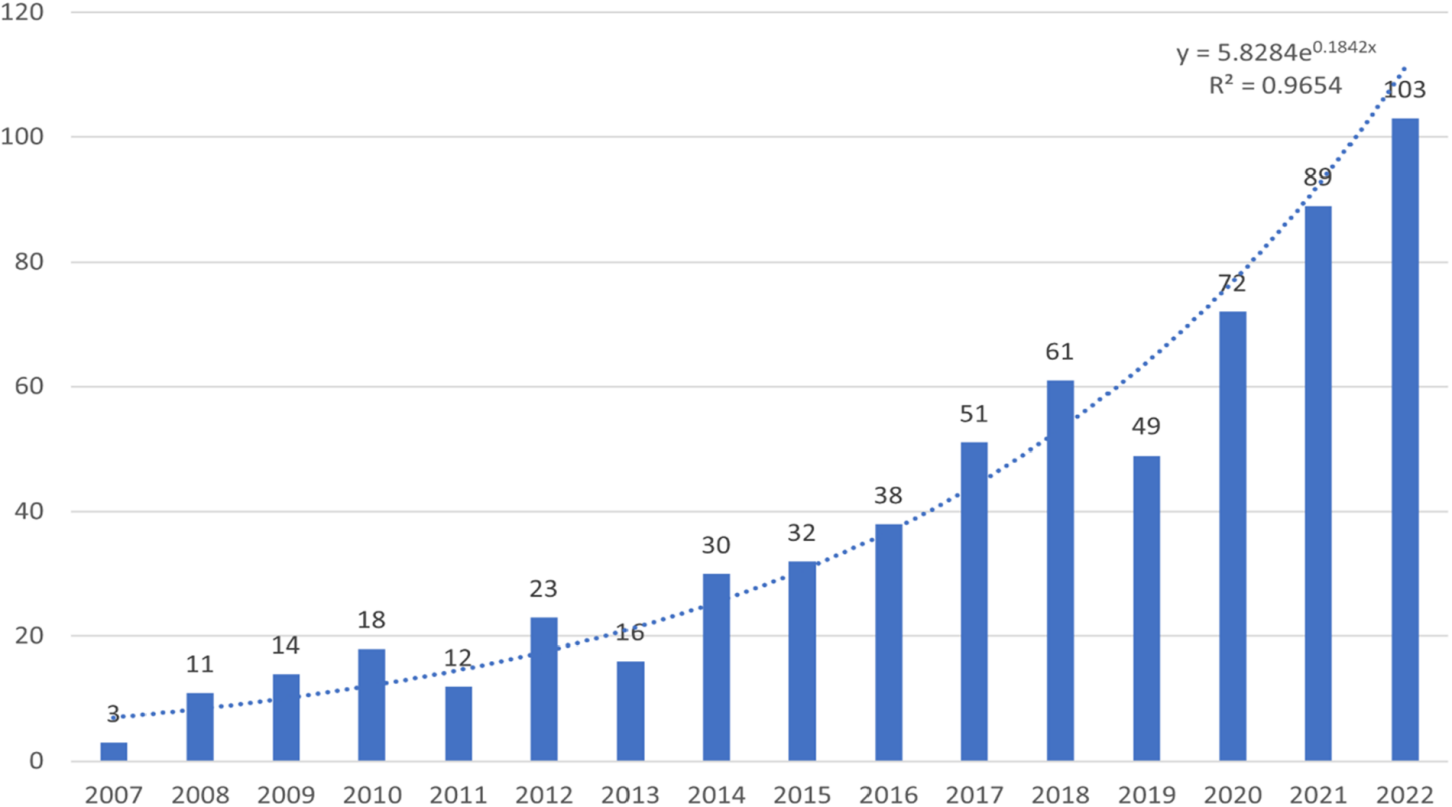
Number of publications per year between 2007 and 2022.

**Figure 3:**
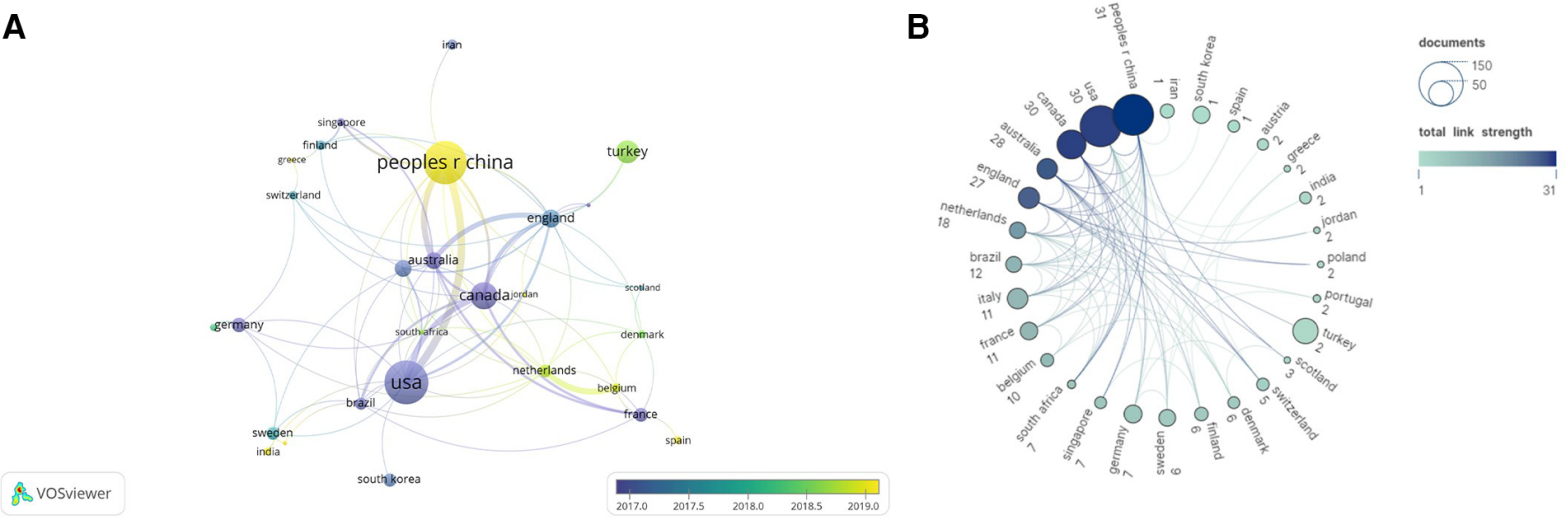
(**A**) Network of countries created by VOSviewer. Node colors indicate when the country appeared. (**B**) Country/regional cooperation network.

**Table 1:** Top 10 countries of preoperative anxiety in children (2007–2022).

Rank	Country	Output	Centrality
1	The United States	121	0.06
2	China	112	0.19
3	Canada	60	0.11
4	Turkey	46	0.00
5	England	32	0.34
6	Italy’	30	0.00
7	Australia	29	0.77
8	Germany	23	0.20
9	France	22	0.06
10	South Korea	21	0.00

As shown in Table 2, the top five institutions are Dalhousie University (15), University of California, Irvine (101), University of Hong Kong (11), Cincinnati Medical Center (11), and Yale University (9). The fact that most of these are US research institutions indicates that the US is a leader in this area of research and plays an important role in the world. Although many academic institutions from other countries publish fewer articles than the United States, these institutions are actively involved in multi-institutional collaborations and still produce many groundbreaking research results.

**Table 2:** Top 10 research institutions for preoperative anxiety in children (2007–2022).

Rank	Institution	Output	Centrality	Original country
1	Dalhousie University	15	0.02	Canada
2	University of California-Irvine	12	0.01	United States
3	The University of Hong Kong	11	0.00	China
4	Cincinnati Children's Hospital Medical Center	11	0.00	United States
5	Yale University	9	0.00	United States
6	HOSPITAL FOR SICK CHILDREN	9	0.00	Canada
7	IWK Health Centre	8	0.00	Canada
8	University of Toronto	7	0.03	Canada
9	Children's Hospital of Orange County	7	0.00	United States

### Bibliometric analysis of authors

**Figure 4:**
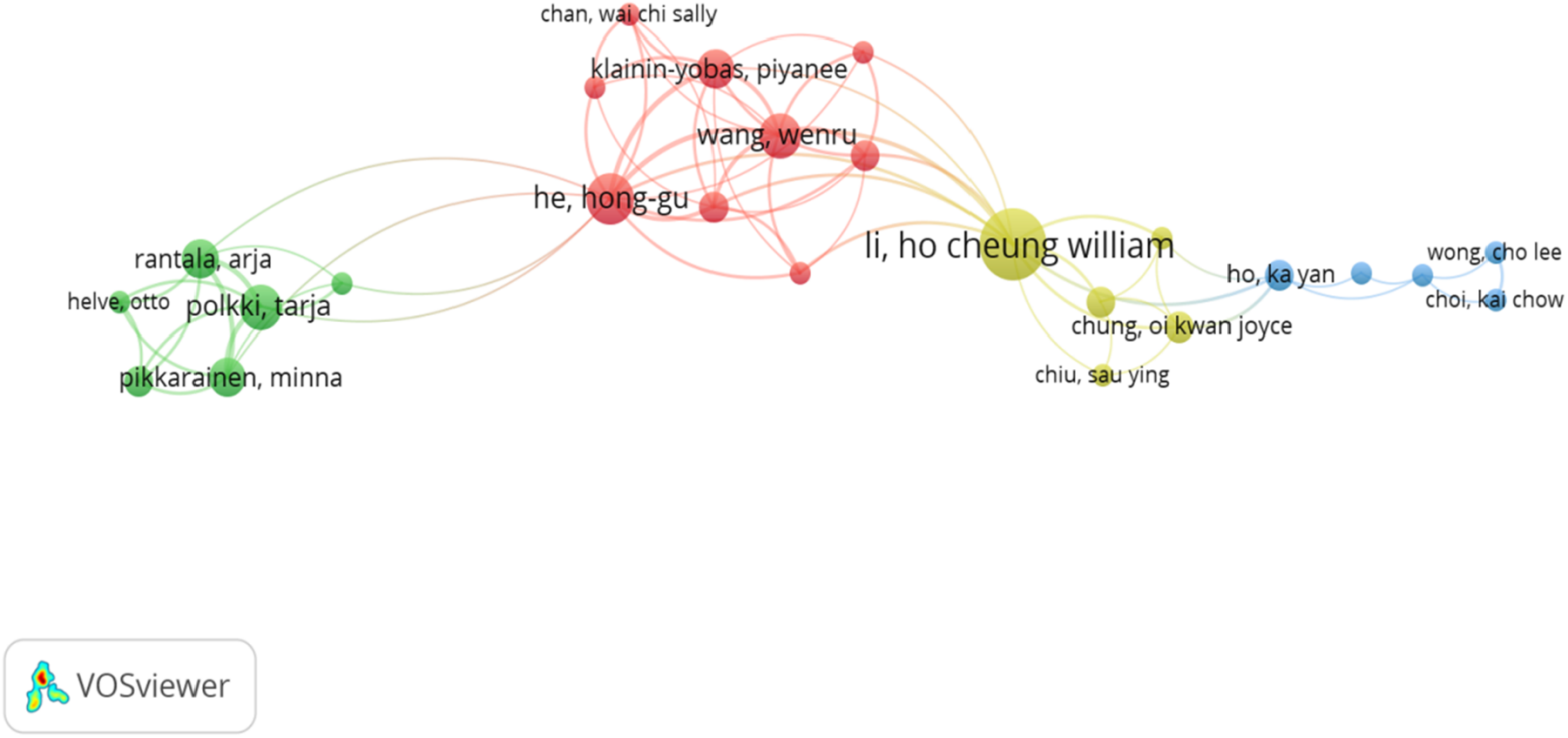
The international cooperation among relevant authors.

Table 3 shows the top 10 authors with the highest number of publications by Kain, Zeev N (13) followed by Fortier, Michelle A (10) and Li, Ho Cheung William (9), and the top five most cited authors are Kain, Zeev N (526), Fortier, Michelle A (427), Li, Ho Cheung William (373), Noel, Melanie (366) and He, Hong-gu (191). Figure 4 shows the author collaboration network generated by VOSviewer. Different nodes represent different authors, the size of the node represents the number of publications by different authors, larger nodes mean more publications by that author, and the thicker the boundaries between different nodes, the more collaboration between authors. As shown in the figure, the relationship between these authors is very decentralized, which means that the scale of collaboration between academics is relatively small. Li, Ho Cheung William, Wang, Wenru, He, and Hong-gu have the three largest nodes, which indicates that they have a great influence on the other authors and can be considered the founding fathers of this study in the field of preoperative anxiety.

**Table 3:** Top 10 authors of preoperative anxiety in children.

Rank	Author	Documents	Citation	Average citation	Centrality’
1	Kain, Zeev N.	13	526	40.46154	0.00
2	Fortier, Michelle A.	10	427	42.7	0.00
3	Li, Ho Cheung William	9	373	41.44444	0.00
4	He, Hong-gu	6	191	31.83333	0.00
5	Mayes, Linda	5	189	37.8	0.00
6	Noel, Melanie	5	366	73.2	0.00
7	Polkki, Tarja	5	17	3.4	0.00
8	Schmidt, Louis A.	5	107	21.4	0.00
9	Staals, Lonneke M.	5	70	14	0.00
10	Van Lieshout, Ryan J.	5	107	21.4	0.00

### Bibliometric analysis of journals

A total of 253 academic journals published 622 research papers related to preoperative anxiety in children. Table 4 presents the top 10 most productive journals. Pediatric Anesthesia [Impact Factor (IF) 2022 = 1.7] published the most papers (55 publications, 8.84%), followed by Journal of Perianesthesia Nursing (IF, 2022 = 1.7; 26 publications; 4.18%), Journal of Pediatric Nursing-Nursing Care of Children & Families (IF, 2022 = 2.4; 22 publications; 3.54%), Anesthesia and Analgesia (IF, 2022 = 5.7; 20 publications; 3.22%) and Journal of Clinical Nursing (IF, 2022 = 4.2; 16 publications; 2.57%). In addition, the top 10 journals included Anesthesia and Analgesia (IF, 2022 = 5.7) and the British Journal of Anesthesia (IF 2022 = 9.8), with an IF above 5. The rest of the top 10 journals had an IF between 1 and 5.

**Table 4:** Top 10 most prolific journals of preoperative anxiety in children.

Rank	Journal	Output (%)	IF (2022)
1	Pediatric Anesthesia	55 (8.84%)	1.7
2	Journal of PeriAnesthesia Nursing	26 (4.18%)	1.7
3	Journal of Pediatric Nursing-Nursing Care of Children & Families	22 (3.54%)	2.4
4	Anesthesia And Analgesia	20 (3.22%)	5.7
5	Journal of Clinical Nursing	16 (2.57%)	4.2
6	Journal of Advanced Nursing	12 (1.93%)	3.8
7	British Journal of Anesthesia	12 (1.93%)	9.8
8	Children-Basel	10 (1.61%)	2.4
9	International Journal of Pediatric Otorhinolaryngology	9 (1.45%)	1.626
10	Frontiers In Pediatrics	8 (1.29%)	3.569

This study uses a dual map visualization to provide a global visualization of literature growth at the discipline level. On the left side of the map is the network of citing journals, with labels representing the disciplines of the citing journals. The network of cited journals is on the right side of the map, with labels representing the disciplines to which the cited references belong. The colored lines starting from the citation map on the left and pointing to the citation map on the right represent the paths of the references.

**Figure 5:**
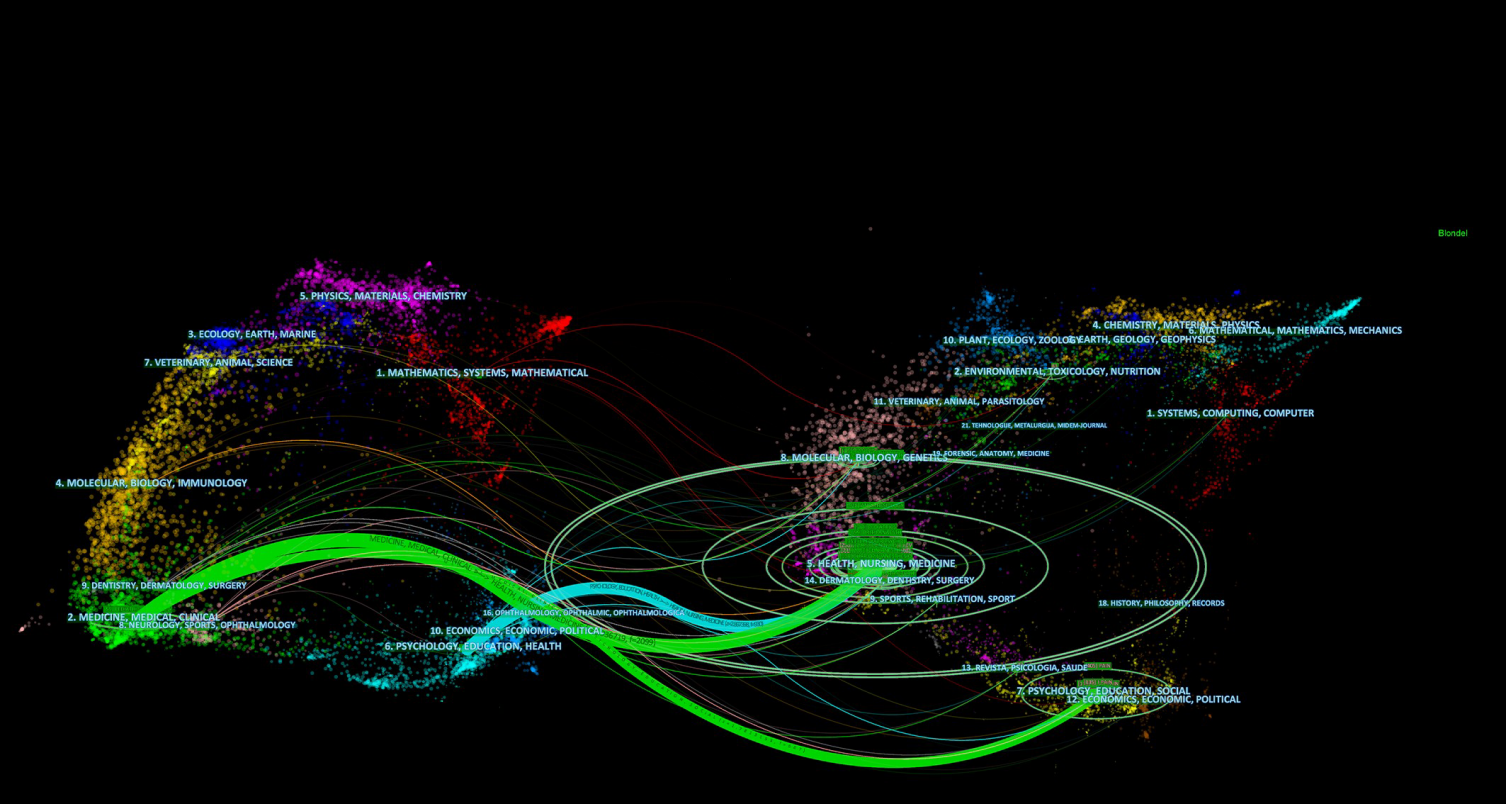
A dual-map overlay of journals.

As presented in Figure 5, the green path represents research published in medicine/medical/dentistry/neurology journals that are typically cited in health/nursing/medicine/dermatology/dentistry/sports/psychology and education journals, and the blue path represents research published in psychology/education/health journals that are typically cited in health/nursing/medicine/dermatology/dentistry/sports journals.

### Bibliometric analysis of keywords

**Figure 6:**
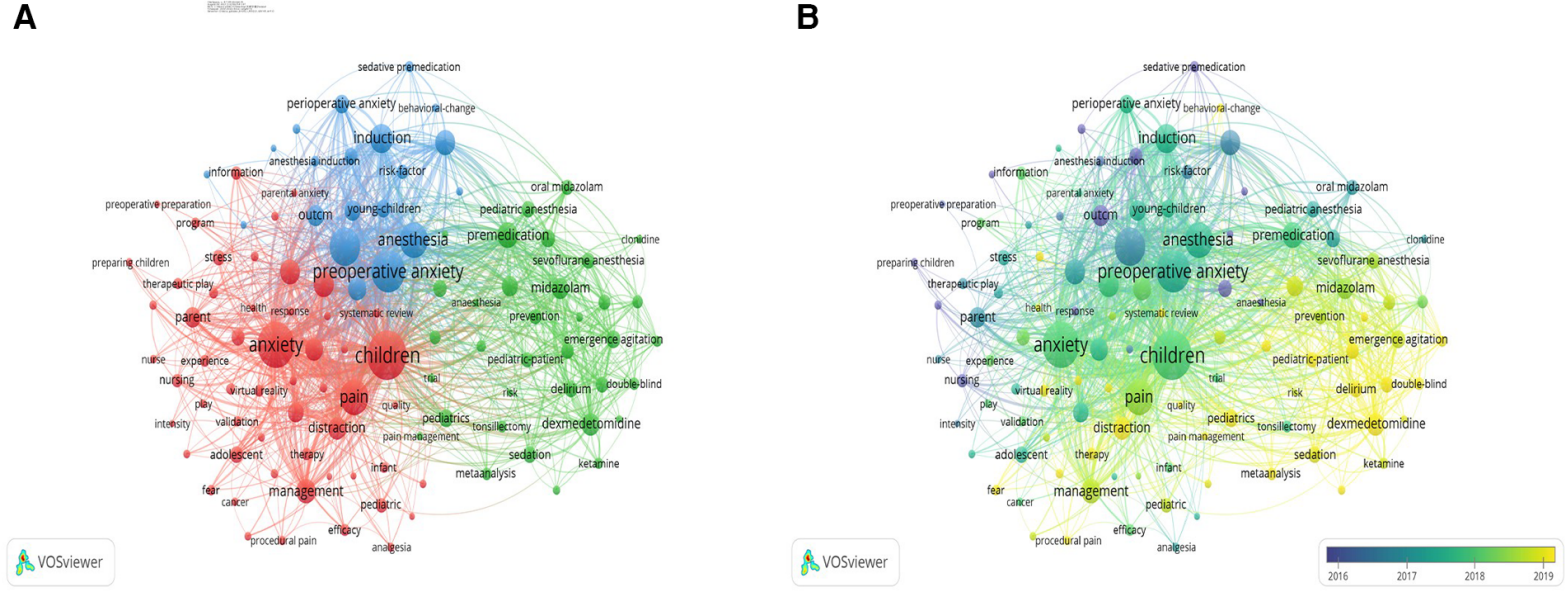
(**A**) Network visualization graph of keyword co-occurrence. (**B**) Chronological keyword distribution based on keyword co-occurrence.

Figure 6 shows a map of the 622 publications analyzed by VOSviewer, including 110 terms (out of a total of 1,980) with at least 10 occurrences of each term; as shown in Table 5 and Figure 6A, the top 10 high-frequency keywords were Children (frequency: 263), Anxiety (frequency: 234), Preoperative anxiety (frequency: 196), Surgery (frequency: 166), Anesthesia (frequency: 148), Pain (frequency: 147), Induction (frequency: 102), Premedication (frequency: 82), Postoperative pain (frequency: 80) and Parental presence (frequency: 79). The most frequent keywords were categorized into three clusters. Cluster 1 (red) mainly refers to nonpharmacological interventions for preoperative anxiety in children, such as “Humor”, “Play”, “Music”, and “Virtual Reality”. Cluster 2 (green) mainly refers to preoperative sedation medications such as “dexmedetomidine”, “midazolam”, “fentanyl”, and “ketamine”. Cluster 3 (dark blue) refers to anxiety risk factors such as “anesthesia induction”, “parental presence”, “young children”, “psychological preparation” and “surgery”. Figure 6B shows the distribution of keywords in chronological order, indicated by the shade of the region's color. Studies before 2016 mostly focused on “Sedative premedication” and “Predictor”; post-2016 studies mostly focused on “Parental presence”, “Surgery”, “Preoperative anxiety”, “Children” and “Anxiety”; and newly discovered research hotspots suggest that “Distraction” and “Dexmedetomidine” seem to be the focus of future research.

**Table 5:** Top 10 keywords of preoperative anxiety in children.

Rank	Frequency	Keywords	Centrality
1	263	Children	0.00
2	234	Anxiety	0.00
3	196	Preoperative anxiety	0.00
4	166	Surgery	0.02
5	148	Anesthesia	0.03
6	147	Pain	0.00
7	102	Induction	0.05
8	82	Premedication	0.04
9	80	Postoperative pain	0.02
10	79	Parental presence	0.05

**Figure 7:**
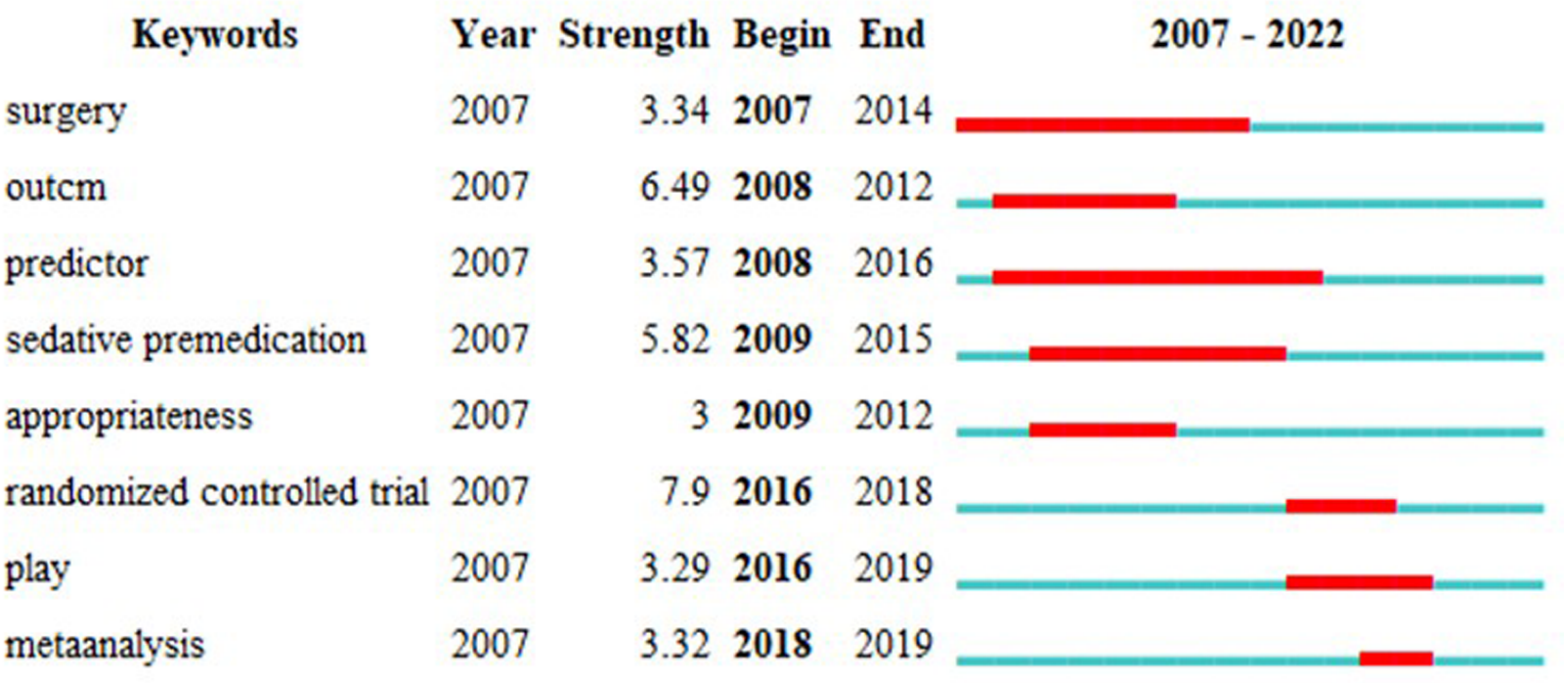
Top 10 keywords with the strongest citation bursts.

Figure 7 lists the ten keywords with the strongest bursts, with the total time span indicated by the blue line and the cycle of bursts indicated by the red line to show the start and end years of the bursts. In 2009–2015, “sedative premedication” had the highest burst intensity (5.82), while “distraction” had the highest burst intensity in 2020–2022 (5.03). These changes over time indicate a shift in research focus. The top three keywords for the strongest bursts are “randomized controlled trial”, “outcm”, and “sedative premedication”. The newest bursts were “distraction” and “sevoflurane”. These are considered the frontiers of research in the field and may be studied in depth and extensively in the future.

### Bibliometric analysis of cocited references

**Figure 8:**
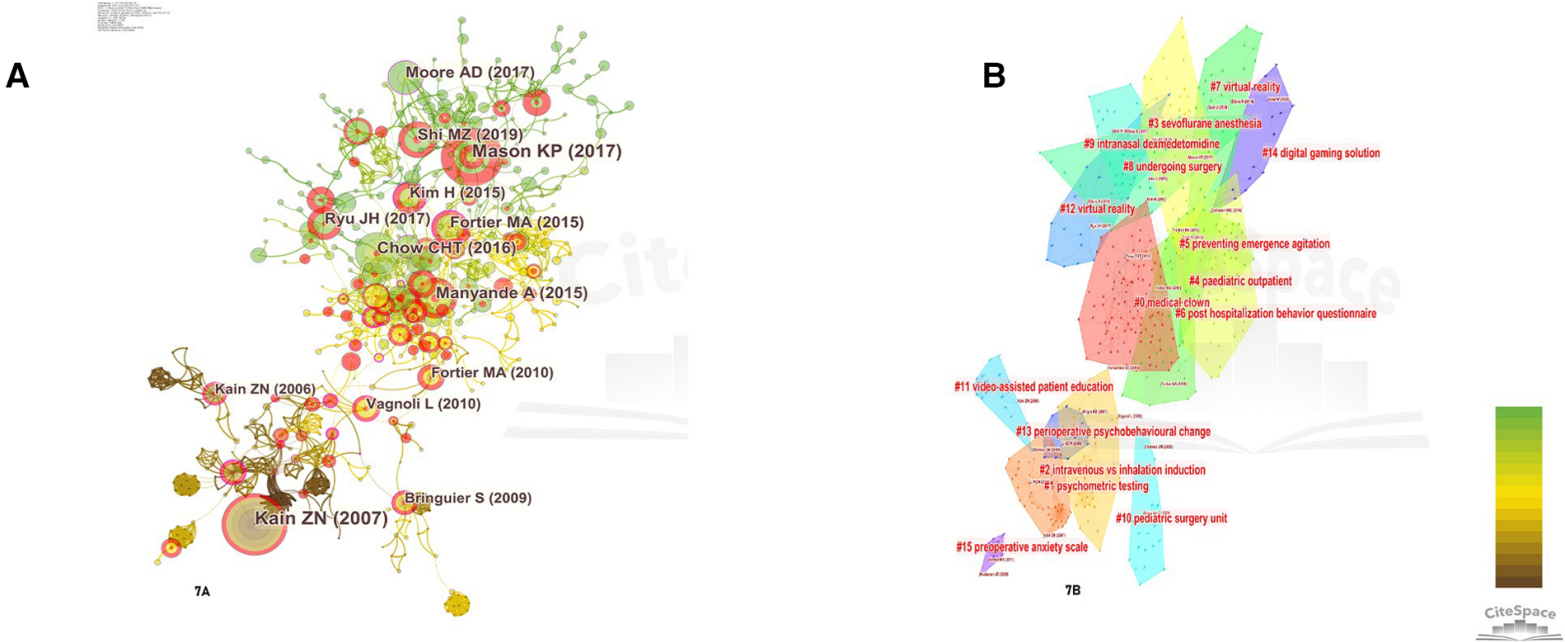
(**A**) The network of cocited references. (**B**) The network of cocited reference clusters.

Cocited references are the theoretical foundation of a research field, and the most cited references can be regarded as hotspots and milestones because they have attracted much attention and made great contributions to the field. Figure 8A is a CiteSpace-generated view of the cocitation network landscape for the period from 2007 to 2022. The network contains 649 nodes and 1,840 links, with each node representing a cited paper and the links between nodes indicating the frequency of citations. The diameter of the nodes is proportional to the frequency of cited references. The different colored regions indicate when the cocited links first appeared in these regions. The color bars range from brown to green, indicating a specific period from 2007 to 2022. For example, green lines or regions generated in 2017 predate yellow or brown lines or regions. Nodes with purple circles represent mediator centrality >0.1. The results of the cluster analysis were convincing for modularity *Q* scores >0.5 and weighted average profile scores >0.7. The network has a modularity *Q* of 0.8657, indicating a well-structured network. The weighted average contour score S is 0.9336, suggesting a strong reliability of the clustering results.

Figure 8B depicts CiteSpace's clustering of the reference cocitation network, showing only the largest 15 clusters based on the index terms and identified by its log-likelihood ratio algorithm. The top five clusters are “medical clown” (#0), followed by “virtual reality” (#1), “anxiety” (#2), “parent” (#3), “emergence delirium” (#4), and “hospital clowning” (#5).

The term “betweenness centrality” refers to the role that nodes play in mediating the transfer of information between other nodes. In this analysis, each node represents a cited reference. References with mediated centrality >0.1 imply that they have a strong connection between two unrelated articles. These references have a high potential to be cited as a knowledge base. They are represented by purple circles.

As shown in Table 6, which demonstrates the top five articles with the strongest mediator centrality, these top five articles were further analyzed, with 1, 3–5 studies focusing on parental anxiety status and the effect of parental presence on preoperative anxiety in pediatric patients and the second article focusing on exploring whether clowning reduces preoperative anxiety in children. Among these articles, Kim H's article had the strongest mediator centrality of 0.35. This study was conducted to determine the effects of video distraction, parental presence, or a combination of the two interventions on reducing preoperative anxiety and postoperative behavioral deficits in preschool children. The results showed no statistically significant difference between the three effects on preoperative anxiety and postoperative behavioral outcomes such as awakening delirium and new onset of negative behavioral changes in preschoolers during induction of inhalation anesthesia, yet suggested two possibilities that, contrary to general opinion, separation from parents may not be the most significant cause of preoperative anxiety in preschoolers. Although children who were accompanied by their parents did not experience separation anxiety, the change in their anxiety levels up to the time of induction of anesthesia was similar to that of children whose parents were not present in the present study, and in fact, the effect of parental presence in transiently lowering children's anxiety has been shown to be only at the time of separation from their parents, not at the time of induction of anesthesia (27). Second, although changes in anxiety levels over time were similar across groups, children with video distraction had lower levels of anxiety at the point in time when they were separated from their parents (transport to the operating room) than those with parents present alone, which likely suggests that parental presence is unlikely to be more effective than video distraction in reducing separation anxiety even in preschoolers, who are the most likely to have a separation anxiety response.

**Table 6:** Five articles with the strongest betweenness centrality.

Article number	Article title	Betweenness centrality	Year	Author
1	Video Distraction and Parental Presence for the Management of Preoperative Anxiety and Postoperative Behavioral Disturbance in Children	0.35	2015	Kim H
	A Randomized Controlled Trial (22)			
2	Clowns for the prevention of preoperative anxiety in children: a randomized controlled trial (23)	0.31	2009	Golan G
3	Parental anxiety and stress before pediatric anesthesia: A pilot study on the effectiveness of preoperative clown intervention (24)	0.29	2014	Agostini F
4	An evidence-based Review of parental presence during anesthesia induction and parent/child anxiety (25)	0.25	2009	Chundamala J
5	Audiovisual aid viewing immediately before pediatric induction moderates the accompanying parents’ anxiety (26)	0.25	2012	Berghmans J

The second study, by Golan G, found that the use of a medically trained preoperative clown on children undergoing surgery significantly reduced preoperative anxiety. However, the clown effect was less pronounced at the time of entry into the operating room and when the anesthetic mask was introduced, and the greatest increase in anxiety was even seen when the anesthetic mask was applied to the child compared to the control group. The study suggests that clown intervention should be limited to the preoperative phase or that further training is needed to use this modality before entering the operating room. The remaining three studies had a centrality of 0.3 or less, with the third study suggesting that clown intervention could positively impact maternal anxiety and stress in the perioperative period and recommending its replication in a clinical hospital setting. The fourth study held a similar view to the first in that parental presence did not appear to significantly alleviate children's anxiety in most cases. These articles serve as critical bridges to suggest areas worth exploring for future research: the relationship between children's and parents’ anxiety states, although tested in studies (28), deserves further exploration, and future randomized trials should measure parents’ and children's anxiety states more objectively and subjectively. A final study has shown that presenting parents with preoperative audiovisual aids during the induction of anesthesia can moderate the increase in parental anxiety associated with the induction of anesthesia.

**Figure 9:**
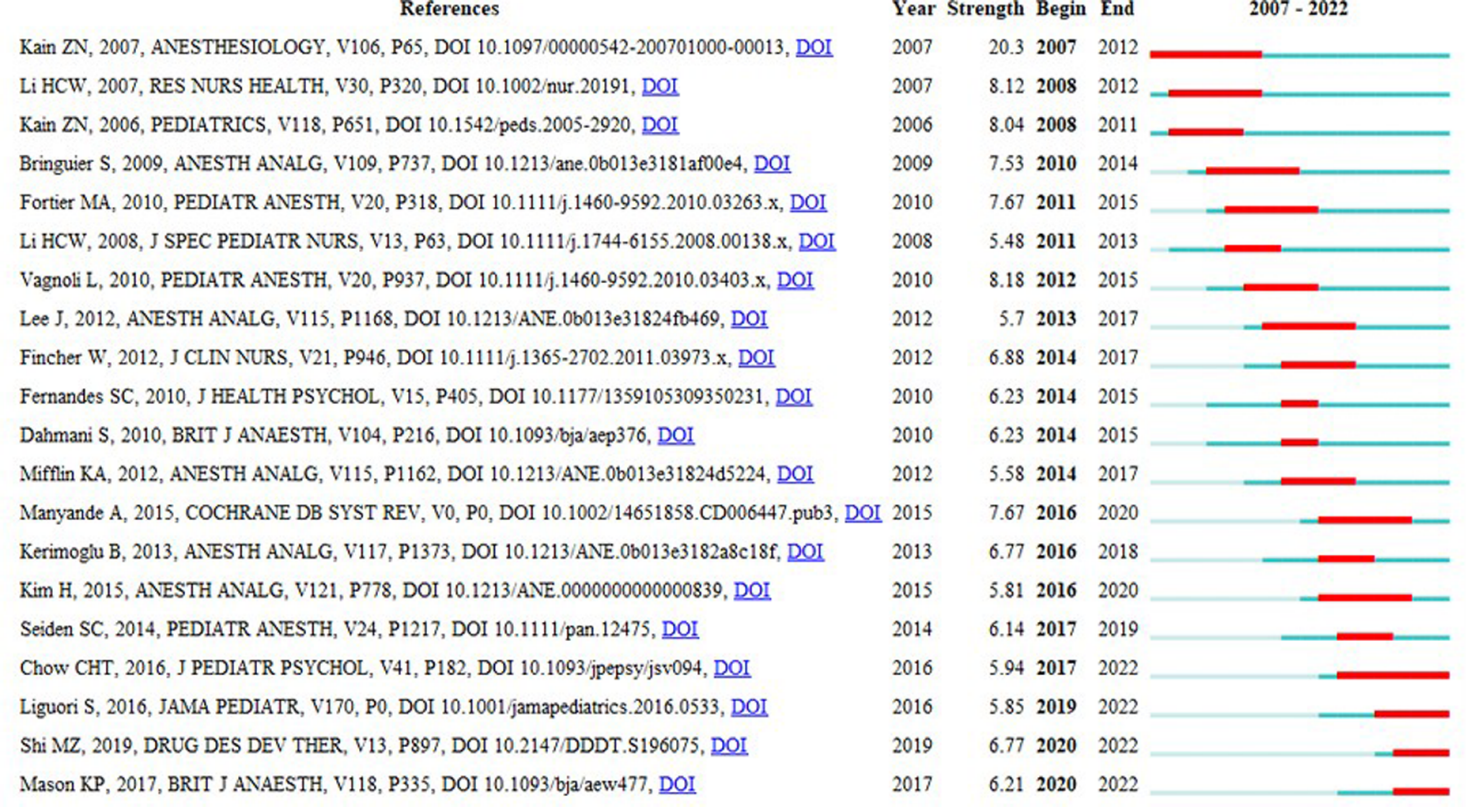
Top 20 references with the strongest citation bursts (2007–2022).

Figure 9 shows the top 20 most frequently cited articles screened using CiteSpace software, and the focus of expert scholars can be obtained by the sudden increase in citation frequency within a short period of time, as shown in Figure 9. There are only four articles with an outbreak intensity of more than 8, which were all published prior to 2015, and the number one cited article is the article on family-centered preoperative preparation to reduce children's preoperative anxiety by Kain ZN (29) (burst intensity of 20.3) and Kain ZN's study in the previous year showing that preoperative anxiety in younger children undergoing surgery was associated with a more distressing postoperative recovery process and a higher incidence of problems such as sleep (3) (burst intensity of 8.04, in fourth place). Vagnoli L's article published in 2010 argues that clowning doctor and parent presence combined intervention was more effective in reducing preoperative anxiety than either sedative medication alone or sedative medication plus parental presence (30) (burst intensity of 8.18, in second place). The third most cited study was by Li HCW (31), which found that preoperative therapeutic play was more effective in reducing children's anxiety levels than preoperative informational preparation alone and in reducing children's negative emotional reactions. Most of these articles describe studies of interventions related to preoperative anxiety in children, with interventions including clown doctors, sedation preoperative medication, family-centered preoperative preparation, and parental presence during induction of anesthesia. There has been a surge in citations in recent years regarding audiovisual interventions such as virtual reality, video distraction, and other electronic technologies (2, 22, 32–34) related to the direction of intervention research, and recent articles based on the citation explosion suggest that the application of more electronic technologies in pediatric surgery to reduce the incidence of preoperative agitation and anxiety will continue to be a hot topic of research in the future, and it is worth noting that there is a large amount of clinical and methodological heterogeneity among the relevant studies It is worth noting that there is considerable clinical and methodologic heterogeneity among the studies (interventions, timing and duration, outcome measures using various scales, different comparison groups, etc.), and it is difficult to definitively state which aspects of the intervention will be most beneficial, and there is a need to further disaggregate these interventions to explore the importance of each separately. In addition, there is a paucity of literature on perioperative anxiety in adolescents, and future research should examine the impact of preoperative anxiety on all children and adolescents undergoing surgery.

## Limitation

The study inevitably has several limitations. First, we subjectively chose a specific search strategy and analyzed only the characteristics of English-language publications retrieved from a single database, WOSCC. This may have caused us to miss some valuable articles from other data archives (e.g., PubMed, Embase, or MEDLINE). Additionally, this study only retrieved English literature, which may have resulted in the omission of important literature in other languages. Furthermore, this study only includes the period 2007–2022, so that the analysis is not up-to-date and not comprehensive enough. Moreover, CiteSpace can be a controversial tool and is constantly being improved. We have only directly described the most popular topics and sketched the general outline of the field as visually as possible; therefore, the depth may not be sufficient, and many details of the articles are not adequately summarized. Most of the results of this study were interpreted by the researcher, which may have led to the analysis being influenced to varying degrees by subjective judgments.

## Discussion

Surgery and hospitalization have been recognized as negative experiences with significant health consequences for adults and young children (35). In particular, it is a very distressing time for hospitalized children, and preoperative discomfort may be associated with postoperative agitation and negative behaviors (36). Stressful events can adversely affect physical and mental health, producing negative outcomes such as mood disorders, cognitive incompetence, and behavioral deficits. Given the potential negative effects of preoperative anxiety, clinicians and researchers have attempted to apply interventions aimed at reducing its prevalence, severity, and impact. These include the use of preoperative sedative medications, psychological preparation programs, and complementary therapies, and given the high number of adverse effects associated with medication as complained of in the introduction, it is crucial to find effective and alternative ways to manage preoperative anxiety in children. The aim of this paper is to provide a detailed bibliometric analysis of studies on preoperative anxiety in children published between 2007 and 2022, incorporating the use of a synthetic knowledge synthesis (37). The analysis reflects the current state of research in the field, lays the groundwork for future studies, and identifies different interventions to reduce preoperative anxiety.

The number of publications on preoperative anxiety in children has increased annually from 2007 to 2022, and after 2017, the articles have all stabilized at more than 40, and this area of research has received increasing scholarly attention in recent years (Figure 2); developed European countries and the United States have dominated this area of research over the past fifteen years. The United States leads the world in terms of the number of studies and publications on preoperative anxiety in children (Table 1), with the University of California, Irvine, accounting for the largest share of research in this area in the future (Table 2). This may be related to its population size and financial commitment. It is noteworthy that most of the top 10 institutions conducting this research are located in Canada and the United States, reflecting the fact that these countries are well equipped to conduct clinical medical research because they provide adequate funding and have state-of-the-art equipment and specialized researchers. In addition, Australia has the highest centrality score and is the country with the most active international collaborations. Surprisingly, the United States appears to have fewer collaborations with other countries and a lower centrality score, despite being ranked first in terms of publications. The centrality of most institutions in the field is less than 0.03, which suggests insufficient collaboration between institutions, and the centrality of the top 10 authors of most publications in the field is less than 0.01. This suggests insufficient collaboration between top researchers. Overall, there is little collaboration between countries, institutions, and authors. Developed countries such as Canada, Germany, Australia, and France started to study preoperative anxiety before developing countries such as China and India, and the University of Hong Kong has become a new institution contributing to preoperative anxiety research in China.

The centrality of the top 10 authors for most publications in the field is less than 0.01, suggesting insufficient collaboration among top researchers. Li, Ho Cheung William; Wang, Wenru; and He, Hong-gu have the three largest nodes, suggesting that they have had a significant impact on the other authors and can be considered the founding fathers of this study in the field of preoperative anxiety.

Journal analysis can provide valuable information to help researchers select relevant journals to present their findings. Our study showed that pediatric anesthesia had the most publications in the field, implying that preoperative anxiety in children is one of the core topics in pediatric anesthesia. We found that the top 10 most prolific journals published less than 35% of the total number of papers, suggesting a significant spread of literature distribution among journals, which may be due to the research direction of the field across multiple fields, covering medicine, dentistry, dermatology, neurology, ophthalmology, psychology, education, health, and others. Since most of the research in this field is interdisciplinary, international collaboration among researchers should be strengthened to produce high-quality research results.

In terms of research hotspots, co-occurring keywords with high frequency can represent various scientific hotspots in the field. They include several key themes: children, anxiety, preoperative anxiety, surgery, anesthesia, pain, induction, preoperative medication, postoperative pain and parental presence. Based on the most frequent keywords, two issues of preoperative children's anxiety were summarized in terms of the following:

(1) The relationship between preoperative anxiety and postoperative pain in children:

Preoperative anxiety and postoperative pain are two important issues faced by children undergoing surgery, and related studies have shown that postoperative pain is higher in children with higher levels of preoperative anxiety (38, 39). Other studies have found that postoperative pain intensity is higher in children with higher levels of postoperative anxiety (40, 41), a phenomenon that may be explained by a study: the physiological mechanisms of pain and anxiety (42). According to the interpretation of Walding, when humans are faced with a stressor, emotional and defense responses are activated, which affects the body's mental system, making the person more alert to his or her surroundings and constantly diverting attention and focusing on his or her surroundings. Therefore, a preoperative reduction in preoperative anxiety in children may be beneficial for postoperative pain relief.

(2) The effect of parental presence during induction of anesthesia on preoperative anxiety in children:

For many, the presence of a parent during the induction of anesthesia intuitively reduces anxiety. However, the effectiveness of parental presence is uncertain, and in some instances (e.g., when the parents themselves have high levels of anxiety), it may increase the child's anxiety, a view supported by Holt and Maxwell (43), who argued that the child's stress is invariably compounded by parental tension and anxiety. An evidence-based review on the effect of parental presence during the induction of anesthesia on preoperative anxiety in children (25) included 11 studies that examined anxiety in children, and the majority of the studies found that parental presence was not more effective than interventions of midazolam, parental presence plus midazolam, or parental presence plus video games. Therefore, it was concluded that contrary to popular belief, parental presence does not appear to reduce anxiety in either parents or children in most cases. However, these findings should be interpreted with caution because the independent variable in previous studies was simply the presence or absence of a parent during induction, and in none of the published studies did the parents make any preparations before being told that they would be accompanying their child into the operating room; more importantly, what exactly the parents did during induction was unknown in these studies. It may be counterproductive for many parents to enter the operating room without significant preparation, and some parental behaviors, such as criticism, excessive reassurance, and commands, may cause additional distress in the child (44). Therefore, the direction of research in this area should be shifted to emphasize the actual behavior of parents during the induction of anesthesia.

The latest buzzwords to break out are “distraction” and “sevoflurane” and these are considered to be the frontiers of research in the field. Using “distraction” as an example, we can predict that one aspect of the future frontier will be the effect of distraction techniques on preoperative anxiety in children; however, there are studies that have shown that the effectiveness of many distraction techniques, such as music and toys, in reducing anxiety during the induction of anesthesia is as yet undetermined (45). Anesthesiologists continue to search for an inexpensive, easy-to-use, and comprehensive approach to reducing anxiety in the pediatric surgical population. Combined with an overall analysis of the outbreak term, we learned that the major research hotspot in this field has shifted from preoperative sedative medications for children to nonpharmacological interventions that distract attention to reduce preoperative anxiety in children, especially audiovisual interventions such as virtual reality (VR), which is the use of head-mounted devices to present a fully immersive three-dimensional environment. A recent systematic review and meta-analysis suggests that VR is effective as a distraction mechanism to reduce pain and anxiety in pediatric patients undergoing medical procedures (7). VR can produce total immersion, i.e., a sense of presence in a virtual environment. Non-virtual reality content, i.e., regular (cartoon) video or 360° video, produces less immersion because the user can only see the filmmaker's movements and the progression of the video (46) This difference in content is important because there is a hypothesis that more immersion is associated with more pain anxiety reduction because less attention is devoted to pain anxiety perception (47) Immersion may also be limited by the need for patients to keep their heads still during certain medical procedures, such as dental treatment. True VR provides the illusion of immersion compared to passive audiovisual glasses and non-VR (360°) videos. Therefore, the role of immersion should be the focus of future research.

In the section on literature co-citations, analyses of the top five most centrally relevant articles and the top five most frequently cited articles, which mostly describe intervention studies related to preoperative anxiety in children, were conducted separately. In recent years, there has been a surge in citations to studies on audiovisual interventions such as virtual reality, video distraction, and other electronic techniques related to the direction of intervention research, and recent articles based on the citation surge suggest that applying more electronic techniques in paediatric surgery to reduce preoperative. While the incidence of agitation and anxiety will continue to be a hot topic for future research, it is worth noting that there is a great deal of clinical and methodological heterogeneity among related studies. Therefore, it is difficult to specify which interventions are most beneficial, and there is a need to further disaggregate these interventions and explore the importance of each intervention separately.

The study of preoperative anxiety in paediatric children has been a hot topic of research in the past and into the future, and interest in preoperative anxiety in children has not diminished, even during pandemics. During pandemics, novel coronavirus pneumonia outbreaks do have socioeconomic, relational, and work-related impacts, all of which can profoundly affect patients’ moods. It has been shown that even though anxiety associated with surgery and anaesthesia did not change significantly during the pandemic, the COVID-19 epidemic has been able to profoundly alter the emotional domain of patients (48). Rates of anxiety and depression have risen dramatically in children and adolescents, not just adults. Although data are limited, the report by Racine et al. shows that during the pandemic, children suffered from anxiety disorders at almost twice the rate of normal children (49). As healthcare professionals who accompany these young patients during the perioperative period, it is important for them to realize that pandemics exacerbate the risk of anxiety, fear, and depression in children. If these issues are not addressed, then children undergoing surgery will experience significant stress, with increased consumption of postoperative pain and analgesic medications, poor sleep, inadequate food intake, and overall maladaptive behavior. Even more challenging, disease control standards require the use of full personal protective equipment (PPE), which leads to depersonalized interactions. As a result, healthcare professionals are unable to use body language and facial expressions as tools to provide reassurance. For future research areas, it is important for healthcare teams to establish a baseline awareness of the dramatic rise in clinical anxiety disorders and depression in order to appropriately change the way pediatric patients are cared for. Early identification of anxiety-prone patients during preoperative screening and planning can help reduce the burden on patients and caregivers.

## Conclusion

We conducted a bibliometric review of the preoperative anxiety literature, analyzing publication trends, hotspots, and research frontiers. Overall, there has been a steady increase in annual production over the past 15 years, and this trend is expected to continue. Findings indicate that the majority of papers are from the United States, with the most productive institutions and authors in terms of number of papers and total link strength being Dalhousie University and Kain, Zeev N. Over time, the research hotspot has shifted from medications for preoperative sedation in children to nonpharmacological interventions for distraction to reduce preoperative anxiety in children. Summarizing future research priorities into three areas: First, emphasizing the actual behavior of parents during induction of anesthesia.Sencond, exploring and emphasizing the role of virtual reality immersion. Finally, the impact of pandemics on preoperative anxiety in children should not be underestimated, and in terms of future areas of research, it is imperative that healthcare teams establish a basic understanding of the dramatic rise in clinical anxiety disorders and depression in order to appropriately alter pediatric patient care practices.
