# Supplementary figures and images for: A bibliometric analysis of research on pediatric preoperative anxiety (2007–2022)
Source: Front Pediatr. 2024 Mar 25;12:1327118. doi: 10.3389/fped.2024.1327118 (PMC10999670; doi:10.3389/fped.2024.1327118)

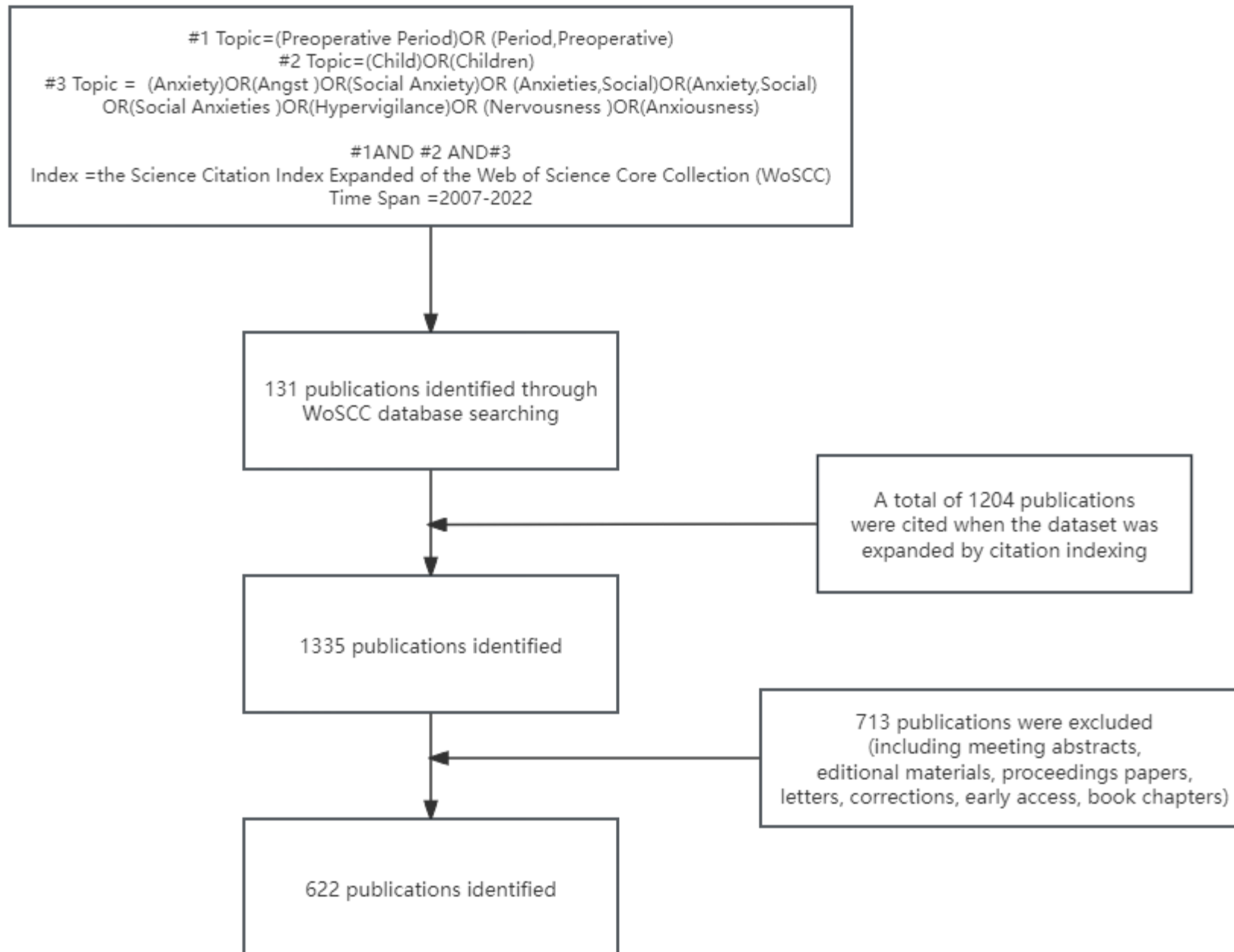

Supplement: Supplementary file 1 [file Datasheet1.pdf]
